# Real-time analysis of gut flora in Entamoeba histolytica infected patients of Northern India

**DOI:** 10.1186/1471-2180-12-183

**Published:** 2012-08-22

**Authors:** Anil Kumar Verma, Ravi Verma, Vineet Ahuja, Jaishree Paul

**Affiliations:** 1 School of Life Sciences, Jawaharlal Nehru University, New Delhi, India; 2 Department of Gastroenterology, All India Institute of Medical Sciences, New Delhi, India

**Keywords:** Gut flora, Entamoeba histolytica, RT-PCR

## Abstract

**Background:**

Amebic dysentery is caused by the protozoan parasite *Entamoeba histolytica* and the ingestion of quadrinucleate cyst of *E. histolytica* from fecally contaminated food or water initiates infection. Excystation occurs in the lumen of small intestine, where motile and potentially invasive trophozoites germinate from cysts. The ability of trophozoites to interact and digest gut bacteria is apparently important for multiplication of the parasite and its pathogenicity; however the contribution of resident bacterial flora is not well understood. We quantified the population of *Bacteroides*, *Bifidobacterium, Ruminococcus, Lactobacillus, Clostridium leptum subgroup, Clostridium coccoides subgroup, Eubacterium, Campylobacter, Methanobrevibacter smithii* and Sulphur reducing bacteria using genus specific primers in healthy (N = 22) vs amebic patients (*E. histolytica* positive, N = 17) stool samples by Real-time PCR.

**Results:**

Absolute quantification of *Bacteroides* (p = .001), *Closrtridium coccoides* subgrou*p* (p = 0.002), *Clostridium leptum* subgroup (p = 0.0001), *Lactobacillus* (p = 0.037), *Campylobacter* (p = 0.0014) and *Eubacterium* (p = 0.038) show significant drop in their population however, significant increase in *Bifdobacterium* (p = 0.009) was observed where as the population of *Ruminococcus* (p = 0.33) remained unaltered in healthy vs amebic patients (*E. histolytica* positive). We also report high prevalence of *nimE* gene in stool samples of both healthy volunteers and amebic patients. No significant decrease in *nimE* gene copy number was observed before and after the treatment with antiamebic drug.

**Conclusions:**

Our results show significant alteration in predominant gut bacteria in *E. histolytica* infected individuals. The frequent episodes of intestinal amoebic dysentery thus result in depletion of few predominant genera in gut that may lead to poor digestion and absorption of food in intestine. It further disturbs the homeostasis between gut epithelium and bacterial flora. The decrease in beneficial bacterial population gives way to dysbiosis of gut bacteria which may contribute to final outcome of the disease. Increase in the copy number of *nimE* gene harboring bacteria in our population reflects possible decrease in the availability of metronidazole drug during treatment of amoebiasis.

## Background

*Entamoeba histolytica*, a micro-aerophilic intestinal protozoan parasite and the causative agent of invasive amoebiasis (colitis and amoebic liver abscess), remains a significant cause of morbidity and mortality in developing countries [1]. It is well known that the parasite is constantly interacting with the intestinal gut flora however the contribution of the flora in the manifestation of the disease is poorly understood. The human gastrointestinal (GI) tract is nutrient-rich environment packed with a complex and dynamic consortia of trillions of microbes [2].The vast majority reside in our colon where densities approach 10^11^ - 10^12^ cells/ml, the highest density recorded for any microbial habitat [3]. About 500–1000 bacterial species colonize the adult intestine,with 30–40 species comprising up to 97% of the total population [4,5]. *Bacteroides, Bifidobacterium, Eubacterium, Clostridium, Peptococcus, Peptostreptococcus, Lactobacillus and Ruminococcus* are considered to be predominant genera whereas *Enterococcus, Methanobrevibacter and sulphur reducing bacteria* (SRB) remain as the subdominant genera [6]. The entire system of the human gut microbiota functions as a ‘microbial organ’ within the intestine, which contributes to diverse mammalian processes including protective functions against pathogens and immune-system modulation, the metabolic function of fermenting non-digestible dietary fiber, anaerobic metabolism of peptides and proteins that results in the recovery of metabolic energy for the host [7]. The microbial diversity of the human gut is the result of co-evolution between microbial communities and their hosts. Microbial community structure is a very important factor that can influence predisposition to specific diseases in certain host contexts [8].

Ingestion of the cyst of *E. histolytica* through fecally contaminated food or water initiates infection. Excystation in the intestinal lumen produces trophozoites and colitis results when the trophozoites penetrate the mucus layer and damages intestinal tissues [9]. The trophozoites proliferate in lumen and phagocytose resident flora. *E. histolytica* trophozoites are quite selective in respect to their interactions with different bacterial species and only those bacteria which have the appropriate recognition molecules get attached and ingested [10]. It has been observed that the nuclear DNA content of *E. histolytica* trophozoites growing in axenic cultures is at least 10 fold higher than in xenic cultures and re-association of axenic cultures with their bacterial flora led to a reduction of DNA content attaining the original xenic values indicating a flexible nature of the parasite genome [11]. Fluctuations in gut flora have been reported both in acute diarrhea and antibiotic associated diarrhea [12], but very few reports are available on status of gut flora in *E. histolytica* infected individuals. Earlier studies in our laboratory [1] have recorded fluctuations in the gut flora by a qualitative method during disease conditions.

5-Nitroimidazole drugs are still used as first line of defense against amoebic and other infections caused by anaerobes. These drugs are administered as pro drugs and one electron reduction of nitro group converts the pro drug into an active drug [13]. Enzymatic modification mediated by *nim*-class of genes is a well characterized resistance mechanism. Certain *Bacteroides* species which are members of the normal colonic human microflora harbor *nim* genes [14]. Our study is based on the hypothesis that the *Entamoeba histolytica* (but not *E. dispar*) is an invasive organism and invades the mucus layer and subsequently the intestinal epithelium for colonization using the pathogenic factors. In this context we attempted to study the fluctuations in the gut microbiota that contributes to substantial metabolic changes in *E. histolytica* infected individuals compared to healthy individuals. In the present study we used Real Time PCR for absolute quantification of predominant gut bacterial population in *E. histolytica* patients suffering from dysentery for 5–7 days. We also quantified the copy number of *nim* gene in stool sample of healthy vs *E. histolytica* patients.

## Methods

### Study subjects & fecal sample collection

Stool samples of healthy person (without any enteric disease) were collected as controls from volunteers of a community in Delhi. Initial survey involved discussion with the focus group and informed consent was taken from participating volunteers for the study. Volunteers in age group of 21–40 year (mean age 31 year) were randomly recruited. Subjects who have taken any antibiotic/antiamoebic drug or suffered from any gastrointestinal disorder in past one month before sample collection were not included in the study. Twenty two stool samples were collected from healthy volunteers. Clinical diagnosis of amoebic colitis was based on standard criteria: patients experiencing days to weeks of dysentery (stool with blood and mucus) or diarrhea with cramps followed by abdominal pain and/or weight loss. The sub acute onset of the disease was a helpful clue in the differential diagnosis because bacillary dysentery caused by Shigella, Salmonella, Campylobacter and EHEC E. coli mostly lead to a abrupt onset of the disease [15]. Since we did not take samples from individuals administered with any antibiotic, therefore cases of antibiotic associated diarrhea were excluded. Stool samples of chronic/acute diarrhea as diagnosed by Gastroenterologist were collected from Gastroenterology department of All India Institute of Medical Sciences & Safdarjung hospitals, New Delhi. The samples were transported to the laboratory at 4°C within 2 hrs and stored at -20°C until processed. The study was approved by the research ethics board of respective institutes. The samples (n = 550) were collected with the informed consent of the patients.

### Enrichment of entamoeba cysts

Cysts were enriched following the protocol of Knight et al., 1976 [16] with slight modifications. Briefly, fecal samples (1gm) were homogenized in 10 ml of autoclaved distilled water, strained through cheesecloth in 50 ml falcon tube. This suspension was centrifuged at 2000 rpm for 5 min and pellet was re-dissolved in 10 ml of 10% formaldehyde. 3 ml of diethyl ether was added to the tube and this mixture was vortexed and incubated at RT for 30 min. The mixture was subjected to centrifugation at 2000 rpm for 5 min, supernatant was removed and pellet was washed with double distilled water. The Pellet containing concentrated cyst was re-dissolved in 400 μl T_10_E_1_ buffer. Cysts in T_10_E_1_ buffer was subjected to freeze-thaw cycle and thereafter to sonication in order to obtain crude DNA for Dot-blot hybridization experiment.

### Screening of samples by dot blot hybridization

**Figure 1:**
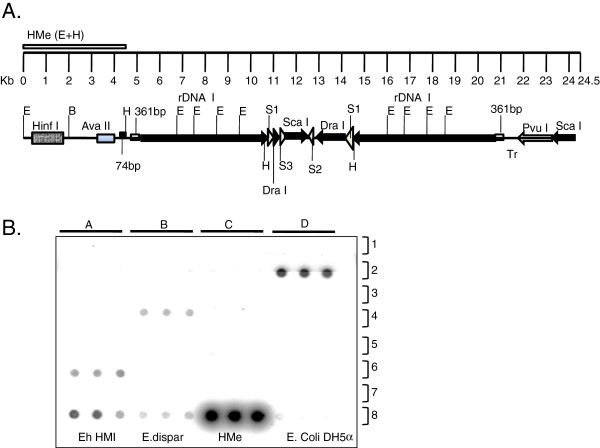
**Screening of stool samples by Dot-Blot method.** (**A**) Linear map of EhRI episome (24.5 kb) showing the position of HMe probe (4.5 kb in size) common for both *E. histolytica* and *E. dispar*), E - EcoR1 site and H- Hind III site; rDNA I and rDNA II represent two inverted repeats of transcription units with various restriction sites and repeats (**B**) Representative figure of Dot-blot analysis of stool sample using HMe probe. Rows 1 to 6 (column A-D) represent spots of DNA from stool samples. About 20 ng of DNA was loaded on each spot in triplicate on nylon membrane. Row 7 was blank. Row 8 (column A) *E. histolytica* HM1: IMSS genomic DNA as positive control; (column B) *E. dispar* SAW760 genomic DNA as positive control; (column C) *E.Coli* DH5α as negative control; (column D) Plasmid with cloned HMe as positive control. All samples were loaded in triplicate. Experimental details are provided in material and methods.

The crude cyst DNA was denatured by addition of NaOH to final concentration of 0.25 N in a total volume of 300 μl. The DNA was kept at room temperature for 30 minutes and then transferred on to ice. The GS + nylon membrane of required size was cut and saturated in 0.4 M Tris-Cl, pH 7.5 for 15 min and the DNA were spotted on to the membrane with the help of mini-fold apparatus from Whatman, Germany. The blots were air dried and UV cross linked before hybridization. We used 4.5 kb rDNA fragment (EcoRI to Hind III site) from HMe region of EhR1 (rDNA plasmid in HM1:IMSS strain of *E.histolytica*) as probe for detection of Entamoeba positive samples that include both *E.histolytica* and *E. dispar* (Figure 1A) [17].

### Genomic DNA extraction

DNA was extracted from the Dot blot positive samples. An aliquot of 200 mg stool sample was used for isolation using QIAamp mini stool kit (QIAGEN,Germany) as per manufacturer’s guidelines. While isolating DNA from the stool samples through the above kit, pGEMT-easy plasmid containg 240 bp fragment of *glycoprotein B* (gB) gene of phocine virus (20 ng/200 μl of ASL buffer) was added in ASL buffer as internal control during the isolation of genomic DNA [18].

### PCR analysis of Dot blot positive samples

**Figure 2:**
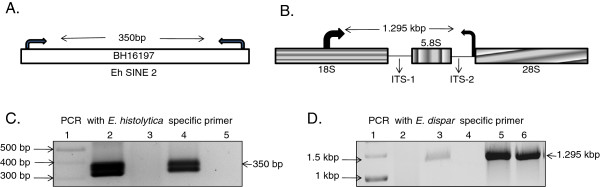
**Screening of Stool samples by PCR.** (**A**) Schematic representation of location of *Entamoeba histolytica* specific primer. BH16197 is Genbank accession number of *Entamoeba histolytica* SINE-2 (EhSINE2) element; (**B**) Schematic representation of location of *Entamoeba dispar* specific primer from rDNA molecule. 18 S, 5.8 S and 28 S are corresponding ribosomal gene sequences and ITS-1 and ITS-2 refers to internal transcribed spacer 1 and 2; (**C**) Detection of *E. histolytica* in stool DNA sample using *E. histolytica* specific primers, Lane 1 = Marker 100 bp, Lane 2 = EhHM1 genomic DNA as positive control, Lane 3 & 4 stool sample DNA, Lane 5 = Genomic DNA of *E. dispar* SAW760 as negative control*.* Sample in lane 4 is *E. histolytica* positive; (**D**) Detection of *E. dispar* in Stool sample using *E. dispar* specific primers. Lane 1 = Marker 1 kb, Lane 2,3,4 and 5 stool sample DNA, Lane 6 = Genomic DNA of *E. dispar* as positive control. Sample in Lane 3 and 5 are *E. dispar* positive. Lane 4 stool sample is *E. histolytica* positive and was used as negative control.

To differentiate Dot-blot positive samples into *E. histolytica* and *E. dispar*, primers were designed from EhSINE2 for *E. histolytica* and from 18 S and ITS2 region of rDNA circle for *E. dispar* respectively (Figure 2A & B)*.* Primer sequences were as follows; Eh-F 5’-GTCAGAGACACCACATGAA-3’, Eh-R 5’-GAGACCCCTTAAAGAAAC -CC-3’ and Ed-F 5’-GAAGAAACATTGTTTCTAAATCCAA-3’ & Ed-R 5’-TTTATTAA CTC ACTTATA-3’ [19].

### Primer designing for detection of predominant genera of gut flora

Primer sets were designed to differentiate and quantitate the following major anaerobic genera –*Bacteroides, Clostridium, Campylobacter, Bifidobacterium*, *Ruminococcus, Eubacterium, Lactobacillus, Methanobrevibacter* and *Sulfate-reducing bacteria* (SRB).16S rRNA gene was targeted for designing primers except for SRB (Table 1). Sulphate reducing gene was targeted for quantifying members of SRB. Primers were commercially obtained from Sigma-aldrich, USA.

**Table 1:** Genus specific 16S rRNA targeted bacterial primers used in this study

**Sr no.**	**Genus**	**Primer sequence**	**PCR Product (bp)**	**Tm(ºC)**	**References**
1.	*Methanobrevibactr*	F 5’- CGATGCGGACTTGGTGTTG-3’	184	59.7	[21]
		R 5’-TGTCGCCTCTGGTGAGATGTC-3’		59.8	
2.	*Peptostreptococcus*	F 5’-AACTCCGGTGGTATCAGATG-3’	270	55.4	[1]
		R 5’-GGGGCTTCTGAGTCAGGTA-3’		56.4	
3.	*Ruminococcus*	F 5’-GAAAGCGTGGGGAGCAAACAGG-3’	302	65.8	[21]
		R 5’- GACGACAACCATGCACCACCTG-3’		64.4	
4.	*Eubacterium*	F 5’-GTAGTCCACGCCGTAAACGATG-3’	278	60.4	[21]
		R 5’-ACACGAGCTGACGACAACCATG-3’		62.4	
5.	*Bacteroides*	F 5’- GGGGTTCTGAGAGGAAG-3’	115	54.0	[21]
		R 5’- GCTACTTGGCTGGTTCAG-3’		56.0	
6.	*Lactobacillus*	F 5’-GCAGCAGTAGGGAATCTTCCA-3’	340	64.0	[25]
		R 5’-GCATTYCACCGCTACACATG-3’		58.0	
7.	*Clostridium leptum subgroup*	F 5’-CGTCAGCTCGTGTCGTGAGAT-3’	125	60.0	[21]
		R 5’-CGTCATCCCCACCTTCCTCC-3’		62.5	
8.	*Clostridium coccoides subgroup*	F 5’-GCCACATTGGGACTGAGA-3’	170	56.0	This study
		R 5’-GCTTCTTAGTCAGGTACCG-3’		58.0	
9.	*Campylobacter*	F 5’-AGGGAATATTGCGCAATGGGGGAAA-3’	180	58.0	[21]
		R 5’- GATTCCGAGTAACGCTTGCACCCT-3’		59.0	
10.	*Bifidobacterium*	F 5’-GATTCTGGCTCAGGATGAACGC-3’	231	61.9	[21]
		R 5’-CTGATAGGACGCGACCCCAT-3’		60.8	
11.	Sulfate-reducing bacteria (APS reductase subunit A gene)	F 5’-TGGCAGATMATGATYMACGG-3’	396	54.	This study
		R 5’-GGCCGTAACCGTCCTTGAA-3’		54.0	

### Primers for detection and quantification of *nim* gene

Primers were designed from *nim* gene after Stephanie Trinh et al. [14]. Primer sequences were as follows; NIM-F (5’-ATGTTCAGAGAAATGCGGCGTAAGCG-3’) and NIM-R (5’-GCTTCCTTGCCTGTCAT GTGCTC-3’). Primers Nim-F and Nim-R designed by us amplify all the members of *nim* gene family viz. *nimA, nimB, nimC, nimD* and *nimE*. Primers were commercially synthesized from Sigma-Aldrich, USA. Primers NIM-F&R did not amplify genomic DNA derived from axenic culture of *E. histolytica* HM1-IMSS [1] and blast result of the selected primers did not show any homology with *E. histolytica* genome. NIM-F&R primers amplified 458 bp fragment of *nim* gene from stool sample DNA. This amplified fragment of 458 bp was cloned in pGEMT-easy vector and sequenced to ensure the amplification of correct gene. The clone was subsequently used as a standard for quantification of *nim* gene by Real Time-PCR.

### PCR-RFLP of *nim* gene

Primers NIM-F and NIM-R were used to amplify all the members of *nim* gene family from stool sample DNA. Members of *nim* gene family were differentiated by digesting the PCR product with restriction enzymes HpaII and TaqI. HpaII digests *nimA, nimC, nimD* at different loci but not *nimB* and *nimE* where as TaqI digests *nimA, nimB, nimE* at different loci but not *nimC* and *nimD*[19].

### Reference strains

Genus specific primers were used to amplify respected genera from DNA of stool sample of healthy individual. The amplified product was cloned and sequenced and sequences were deposited in EMBL database to obtain the accession numbers (Table 2).These 16S rRNA gene fragment containing plasmids were used as reference strains.

**Table 2:** Accession number of reference strain used in the study

**Bacteria**	**Source**	**Accession no.**
*Bacteroides*	Stool of healthy individual	AM117604
*Methanobrevibacter*	Stool of healthy individual	FN813615
*Eubacterium*	Stool of healthy individual	FN813614
*Lactobacillus*	Stool of healthy individual	AM042701
*Bifidobacterium*	Stool of healthy individual	AM042698
*Clostridium*	Stool of healthy individual	AM042697
*Campylobacter*	Stool of healthy individual	AM042699
*Ruminococcus*	Stool of healthy individual	FN823053
*Sulfate-reducing bacteria*	Stool of healthy individual	FN995351

### Real time PCR analysis of bacterial population

Quantification was done using ABI-7500 machine and power syber green PCR master mix kit from Applied Biosystems, USA. Standard curve was the method of choice for absolute quantification of bacteria. Standard curve was made using serial dilutions of plasmid (containing 16S rRNA gene fragment) of known concentrations on tenfold basis. With the molecular weight of the plasmid and insert known, it is possible to calculate the copy number as follows:

Step 1: Determining molecular weight (mw)

Weight in Daltons (g/mol) = (bp size of double stranded product)(330 Da x 2nt/bp)

Step 2: Molecular weight to copy number

X g/mol**/**Avogadro’s number (6.023 × 10^23^ molecules/mole) = X g/molecule

Where X = the weight of one molecule or copy

Where bp = base pairs, nt = nucleotides [20]

Real time PCR runs were performed in 96 well optical plates (each containing 1x PCR master mix, 4 pm/μl forward and reverse primer(optimized concentration) and 1μl plasmid DNA of tenfold dilutions or 1μl DNA from samples in 20μl reaction) for 40 cycles using an ABI 7500 sequence detector (Applied biosystems). Default 7500 cycle conditions were used with only change in the annealing temperature. A standard curve was drawn by plotting the natural log of the threshold cycle (Ct) against the natural log of the number of molecules. Melting curves were obtained from 55°C to 90°C, with fluorescence measurements taken at every 1°C increase in temperature. All reactions were carried out in triplicate along with a non-template control. Ct values were calculated under default settings for the absolute quantification using the software provided with the instrument. The equation drawn from the graph was used to calculate the precise number of target molecule (plasmid copy no. or number of bacteria) tested in same reaction plate as standard as well as in sample.

### Statistical analysis

Graph of respective bacterial population is plotted as mean value with standard error. Each sample was analyzed in triplicate for calculation of significant differences in bacterial population by the Man-Whitney test. P values of 0.05 or below considered as significant. Paired samples collected from healthy volunteers before and after satronidazole treatment were analyzed by Wilcoxon matched-pairs signed rank test (two tailed). Analysis was done using GraphPad Prism-5 software.

## Results

### Screening of *E. histolytica* positive samples

DNA from concentrated cyst was subjected to Dot-blot hybridization. Dot blot analysis of 550 samples yielded 39 samples (7%) that were positive for Entamoeba (Figure 1B). The DNA from Entamoeba positive samples were subjected to PCR using species specific primers of *E. histolytica* and *E. dispar* (Figure 2C & D)*.* Out of 39 samples, 17 samples (43%) were positive for *E. histolytica*. None of the samples in our study population were found positive for both the species of the parasite.

### Quantification of predominant flora

**Figure 3:**
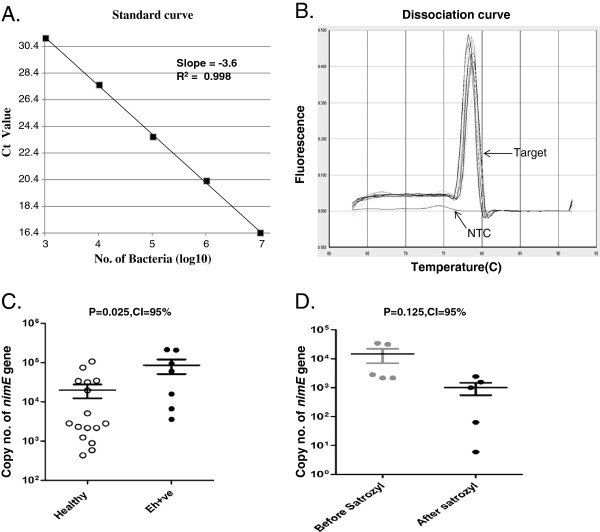
**Real-time analysis for quantification of different bacterial genera in Healthy vs *E. histolytica *positive (Eh + ve) samples.** (**A**) *Bacteroides* (**B**) *Clostridium coccoides subgroup* (**C**) *Clostridium leptum subgroup* (**D**) *Lactobacillus* (**E**) *Campylobacter* (**F**) *Eubacterium.* P value = .05 or below was considered significant. CI stands for confidence interval.

High quality DNA isolated from *E. histolytica* positive stool sample was subjected to Real Time analysis to assess the predominant gut flora that included *Bacteroides, Bifidobacterium, Eubacterium, Clostridium leptum* subgroup*, Clostridium coccoides* subgroup*, Lactobacillus and Ruminococcus.* Two subdominant genera *Methanobrevibacter smithii* and Sulphur reducing bacteria (SRB) were also quantified. Validation of primers designed by us for the above genera have already been reported [21]. In addition to the above primers, here we report a Real time analysis of *nim* gene copy number for which a standard curve and amplification curve have been drawn that shows specific and efficient quantification with slope = −3.6 and R^2^ =0.998 (Figure 3A & B).

**Figure 4:**
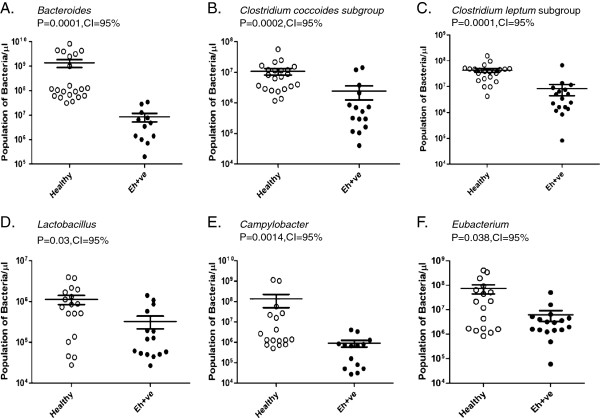
**Real-time analysis of population of (A) Rumminococcus in Healthy vs *E. histolytica *positive (Eh + ve) samples (B) Bifidobacterium in Healthy vs *E. histolytica *positive (Eh + ve) samples.** P value = .05 or below was considered significant. CI stands for confidence interval.

**Figure 5:**
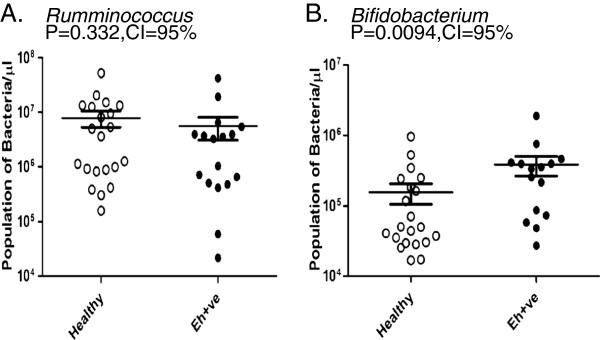
**Detection and identification of *nim *gene in stool samples.** (A) Detection of *nim* gene using *nim* gene specific primers. Lane 1 = Marker 100 bp, Lane 2 = clone of *nim* gene as positive control, Lane 3–5 = DNA from stool samples from healthy volunteer, Lane 6–8 = DNA from stool samples from *E. histolytica* positive patients and Lane 9 = No template control PCR (**B**) Restriction map of TaqI restriction sites in 458 bp *nimE* gene fragment. (**C**) HpaII does not digest *nimE*,where as digestion of *nimE* by TaqI generates four fragment of 274 bp,155 bp,6 bp and 25 bp. Lane 1 = Marker 100 bp, Lane H1, H2, E1 and E2 show RFLP profile of PCR product digested with HpaII; Lane H3, H4, E3 and E4 show RFLP profile of PCR product digested with TaqI. H1-H4, DNA from stool samples of Healthy volunteers and E1-E4 are DNA from stool samples of *E. histolytica* positive patients.

Our analysis reveals that during healthy conditions, the members of *Bacteroides* were the most abundant in number among the predominant targeted genera. However, a significant decrease was observed in population of *Bacteroides* (p = .001) in *E. histolytica* positive samples when compared to that of Healthy control samples (Figure 4A). Simultaneously, we also observed a significant decrease in the population of *Closrtridium coccoides* subgrou*p* (p = 0.002), *Clostridium leptum* subgroup (p = 0.0001), *Lactobacillus* (p = 0.037), *Campylobacter* (p = 0.0014) and *Eubacterium* (p = 0.038) in *E. histolytica* positive samples in comparison to control (Figure 4B, C, D, E and F respectively). Surprisingly, we observed a significant rise in the population of *Bifidobacterium* (p = 0.009) in amebic samples when compared with healthy control samples (Figure 5B). No significant changes were observed in population of *Rumminococcus* (p = 0.33) (Figure 5A). Though we did not observe any significant change in the population of *Methanobrevibacter* (p = 0.96) and Sulphur reducing bacteria (p = 0.88) in amoebic samples but the prevalence rate was reduced (Additional file 1: Figure S1A & B).

### Copy no. of *nim* gene

**Figure 6:**
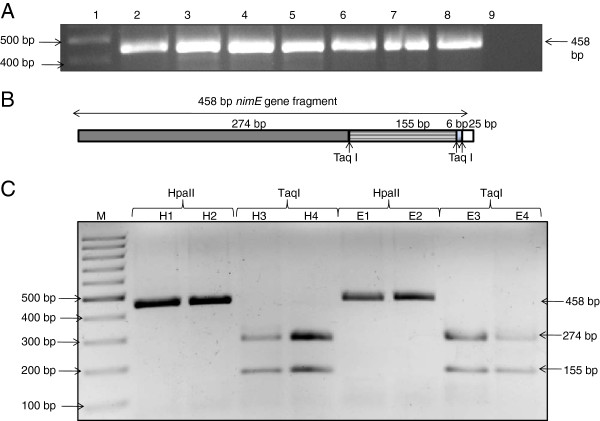
**Detemination of copy number of *nimE *gene by Real-time PCR.** (A) Standard curve, slope = −3.6 and R^2^ = 0.998 showing good efficiency. (**B**) Dissociation curve showing specific amplification of target (*nimE* gene) and NTC = No template control. (**C**) Absolute quantification of copy no. of *nimE* gene in Healthy vs *E. histolytica* positive samples. (**D**) Absolute quantification of copy no. of *nimE* gene in stool sample DNA of Healthy volunteers before and after satronidazole treatment. P value = .05 or below was considered significant. CI stands for confidence interval.

We found the presence of *nim* genes in 72.7% of control stool samples (n = 22) and in 41% of *Entamoeba histolytica* infected patients (n = 17) by PCR (Figure 6A). Further the amplified product was cloned and sequenced. BLAST analysis revealed 99% sequence homology with *nimE* gene (Accession no. AM117602.1), a member of *nim* gene family [22]. Subsequently, the PCR products from all the samples of healthy and amebic individuals were subjected to RFLP analysis using HpaII and TaqI restriction enzymes. PCR-RFLP pattern confirmed the presence of only *nimE* gene in all the samples analyzed (Figure 6B & C). Real time analysis of *nim* gene in the stool samples exhibited sample to sample variation (4 × 10^2^ to 4 × 10^5^ copies) in the both category of samples. We observed a significant increase in copy no. of *nim* gene in *E. histolytica* positive samples vs samples from healthy persons (p = 0.025) (Figure 3C).

To see the effect of antiamoebic drug Satronidazole (Alchem pharmaceuticals) on *nim* gene copy number, healthy volunteers (n = 5) were advised to take the drug (300 mg tablets) twice daily after meals for 4 days and copy of *nim* gene was quantified before and after the treatment using the primers described here. Wilcoxon matched-pairs signed rank test (two tailed) analysis of copy no. of *nim* gene shows no significant change (p = 0.125) in stool samples collected before and after treatment (Figure 3D).

## Discussion

Infection by *E. histolytica* is normally initiated by the ingestion of fecally contaminated water or food containing *E. histolytica* cysts. Phagocytosis of colonic bacteria has been considered as a possible stimulus to induce the invasive behavior by the parasite [23]. Adult gut microbiota are quite stable in individuals and can even be restored after perturbation [24,25]. Our earlier results have shown significant changes in expression of EhCaBP and LPG only after the axenic *E. histolytica* had been adapted to grow with bacterial flora for a number of generatiom, and not in short term culture [26]. In the present study we tried to evaluate perturbations in commensal gut flora caused as result of *E. histolytica* infection using Real Time PCR. qPCR methodology is less expensive, more quantitative and is more efficient in terms of time and operation [27]. The absolute proportions of eight predominant commensal and two subdominant genera were quantified successfully in our samples.

*Bacteroides* species are a pleomorphic group of non-spore forming gram-negative anaerobic bacteria. Bacteroides are the most dominant part of the normal indigenous flora in the human gut. *Bacteroides* are mostly represented by *Bacteroides ovatus, Bacteroides uniformis Bacteroides vulgatus*, *Bacteroides thetaiotaomicron*, *Bacteroides distasonis*, and less frequently by *Bacteroides eggerthii* and *Bacteroides fragilis.* These bacteria are significant contributors to the carbohydrate metabolism, nutrition and health of humans and animals. In 1999 Hooper et al. demonstrated that *B. thetaiotaomicron* can modify intestinal fucosylation in a complex interaction mediated by fucose repressor gene and a signaling system [28]. The significant decrease in population of *Bacteroides* during disease condition dampens the beneficial effects of this genera to host.

In the human intestinal tract, *Eubacterium* is the second most common genus after the genus *Bacteroides.* The genus *Eubacterium* comprises a nutritionally diverse group of organisms. The members of genus *Eubacterium* are known to produce butyrate [29], degrade flavonoids (from vegetables, fruits, nuts, and tea) [30] and are implicated in steroid and bile transformation in intestine [31]. The decrease in population of *Eubacterium sp.* observed in our study may reduce the butyrate production and may also affect the capacity of the host in proper digestion of the above ingredients of food*.*

*Bifidobacterium* species are common inhabitants of the gastrointestinal tract, and they have received special attention because of their health-promoting effects in humans. Members of *Bifidobacteria* produce enough acetate (SCFA) in proximal and distal colon by fermentation of glucose and fructose [32]. Members of both *Bifidobacteria* and *Ruminococcus* -*Ruminococcus torques* and *Bifidobacterium bifidum* are thought to ferment mucin and compete to colonise this substrate for their energy source [33]. Our result shows a significant increase in population of *Bifidobacterium* but no change in population of *Rumminococcous* despite decrease in population of several other targeted genera. It is quite well known that mucus secretion is increased in *E. histolytica* infection especially during dysentery which is probably result of a mechanism exerted by intestinal epithelial cells to counter the adherence of *E. histolytica* trophozoites to intestinal epithelial surface. The protozoan parasite *Entamoeba histolytica* cleaves Mucin 2 (MUC2) in the non-glycosylated oligomerization domains by cysteine protease, thus breaking down the macromolecular structure and reducing mucus viscosity [34]. Perhaps under this condition, a cross-talk between the mucosal layer, bacteria and the parasite initiates. As a result, the intestinal epithelial cells tend to produce more of mucin for protection that promotes colonization of *Bifidobacteria* in one hand and on the other hand the parasite competes to more release of mucin for its adhesion to epithelial layer. *Bifidobacteria longum* are known to protect the gut from enteropathogenic infection through production of acetate [32] and acetate is major energy source for colonocytes but a fine balance in population of different bacterial genera of gut is needed for healthy colon.

The *C. leptum* subgroup and *C. coccoides subgroup* are one of the most predominant populations of human fecal microflora which contains a large number of butyrate-producing bacteria [35,36]. Butyrate is a SCFA (Short chain fatty acids) having a strong effect on the cell cycle and acts as anti-inflammatory molecule in the gut. Effects on mucosal defense include improved tight junction assembly, antimicrobial secretion and mucin expression [37]. The decrease in population of members of *C. leptum* subgroup and *C. coccoides* subgroup observed here leads to decrease in the production of SCFA and hence renders the host more susceptible for future infections.

The genus *Lactobacillus* comprises a large heterogenous group of low G + C gram positive, non sporulating, anaerobic bacteria belonging to phylum Firmicutes. *Lactobacilli* are known to fortify epithelial barrier by various mechanism such as induction of mucin secretion, enhancement of tight-junction functioning, upregulation of cytoprotective heat shock proteins and prevention of apoptosis of epithelial cells [38]. Probiotic strains of *Lactobacillus* are known to prevent infectious diarrhea, antibiotic associated diarrhea and diarrhea in children who are unusually more susceptible as a result of poor nutrition, impaired immune status or frequent exposure to pathogens [39]. We observed significant decrease in population of *Lactobacillus* in gut flora of *E. histolytica* positive patients as compared to that of healthy individuals that support our earlier observation made by semi quantitative method [1].

*Methanobrevibacter smithii* is the dominant archaeon in human gut that affects the specificity and efficiency of bacterial digestion of dietary polysaccharides, thereby influencing host calorie harvest and adiposity [40]. It has been suggested that the low and variable prevalence of *Methanobrevibacter smithii* and *Methanosphaera stadtmanae* DNA in human stool contrasts with the paramount role of these methanogenic archaea in digestion processes and hypothesized that this contrast is a consequence of the inefficiencies of current protocols for archaea DNA extraction [41]. In our samples prevalence of *M. smithii* in healthy individuals stool samples was 27.27 % and that was further reduced to 11.7 % in *E. histolytica* positive samples. Real-time analysis shows no significant alteration in population of *M. smithii*. Variation in the loads of *M. smithii* under different pathophysiological condition such as during amebiasis has not been reported so far.

Suphate reducing bacteria (SRB) are a group of non spore forming, gram negative, dissimilatory sulphate reducing, anaerobic bacteria. SRB can be isolated from the intestinal tract of humans and various environmental sources. Intestinal SRB’s growth and resultant hydrogen sulfide production have been implicated to damage the gastrointestinal tract and thereby contribute to chronic intestinal disorders [42]. *Desulfovibrio fairfieldensis* and *D. desulfuricans* have been associated with incidence of bacteremia and *D. vulgaris* has been associated with intra-abdominal infections [43]. The prevalence of Sulphate reducing bacteria was 36.36% in healthy and 11.7% in amoebic individuals stool samples. However, the change was not statistically significant.

The genus *Campylobacter* is notorious for causing gastroenritis by *C. jejuni* but uncultured *Campylobacter species* e.g. *Campylobacter hominis* whose role is not clear yet, do exist in lower gastrointestinal tract of healthy humans [44]. We observed significant decrease in population of *Campylobacter* in *E. histolytica* positive individual as compared to healthy individuals. As our primers were genus specific, so decrease in *Campylobacter* was genera specific and not species specific. Significant increase in the population of *Campylobacter* has been observed in IBD [21] but we did not find the same trend in amoebic patients.

Several species of *Bacteroides* are known to harbor *nim* genes e.g. *B. fragilis, B. distasonis, B. thetaiotaomicron, B. vulgatus, B. ovatus* but wide differences in MIC values of metronidazole are observed, ranging from 1.5 to >256 mg/L and some are also found above the therapeutic breakpoint of 16 mg/L [45].Though the population of *Bacteroides* is decreased significantly in *E. histolytica* positive patients however we have observed high copy no. of *nimE* gene in the same. We attribute this increase to the presence of plasmid coded *nimE* gene as has been observed earlier in Veillonella sp. [46]. Future analyses that target specific members of the *Bacteroides* group will shed further light on the species involved in the expansion of *nimE* gene**.** In 2006, Rani et al. reported presence of *nim* gene in stool samples of amebic individuals but not in healthy individuals [1] but our result show high prevalence rate of *nim* gene even in healthy individuals irrespective of the disease. However in a hospital based study carried out in Greece revealed low level of prevalence of *nim* gene in isolates of different anaerobic bacterial species from hospitalized patients [47]. Though the presence of *nim* gene in gut of healthy north Indian population is shocking but this may be explained due to easy over the counter drug availability in India. Results on healthy individuals undergoing Satronidazole treatment indicate that *nimE* gene copy number does not show significant reduction. It can therefore be assumed that *nimE* gene harboring *Bacteroides* probably cause inactivation of nitroimidazole drug and thereby reduce the bioavailability of drug to the parasite and hence may help in sustaining the infection.

## Conclusion

The metabolic activities of the predominant gut flora have a significant effect on the health of the human colon. The current findings of depleted populations of metabolically important bacteria like *Bacteroides, C. leptum* and *C. coccoides* sub groups, *Lactobacillus sp*., *Eubacterium sp.,* and *Campylobacter sp.* add to our knowledge of the changes in the GI tracts of amebic patients. Such changes in bacterial population in the normal microbiota could have considerable consequences in terms of functional potential of gut flora and could result in metabolic conditions favorable for the establishment of opportunistic pathogens (e.g. *Clostridium difficile*). However, our study cannot conclude that observed changes in the gut flora is the cause or effect of the infection or the effect of dysenteric mechanism *per se* by the parasite. Our findings could potentially guide implementation of dietary/probiotic interventions that impact the gut microbiota and improve GI health in individuals infected with *Entamoeba histolytica*.

## Abbreviations

SRB: Sulphur reducing bacteria; RFLP: Restriction fragment length polymorphism; MUC2: Mucin 2; SCFA: Short-chain.

## Competing interests

We declare that no competing interests exist among the authors

## Authors’ contributions

JP conceived and coordinated the study. AKV carried out the bacterial quantification experiments. AKV and RV conducted the copy number calculation experiments. JP and AKV drafted the manuscript and conducted the statistical analysis. VA made the diagnosis of the patients, interpretation of data and collaborated in collection of the samples. All authors read and approved the final manuscript.

## Supplementary Material

Additional file 1 **Real time analysis of population of (A) Methanobrevibacter in Healthy vs E. histolytica positive samples (B) Sulphur reducing bacteria in Healthy vs E. histolytica positive sample. P value = .05 or below was considered significant.** Cl stands for confidence interval. Click here for file
