# Supplementary material for: Real-time analysis of gut flora in Entamoeba histolytica infected patients of Northern India
Source: BMC Microbiol. 2012 Aug 22;12:183. doi: 10.1186/1471-2180-12-183 (PMC3534334; doi:10.1186/1471-2180-12-183)
Supplement: Additional file 1 — Real time analysis of population of (A) Methanobrevibacter in Healthy vs E. histolytica positive samples (B) Sulphur reducing bacteria in Healthy vs E. histolytica positive sample. P value = .05 or below was considered significant. Cl stands for confidence interval. [file 1471-2180-12-183-S1.pptx]

## Slide 1
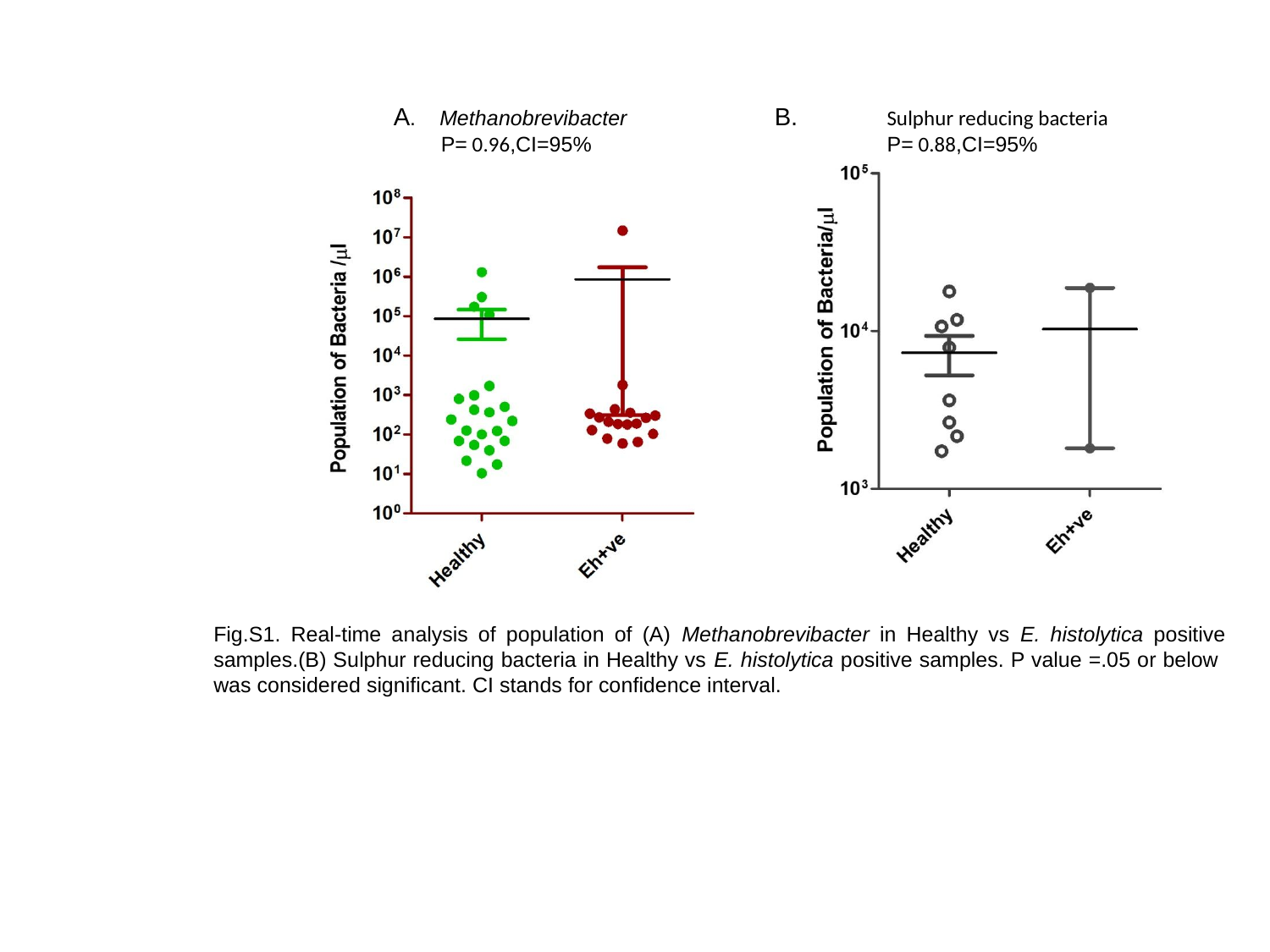

A. Methanobrevibacter
 P= 0.96,CI=95%
B. Sulphur reducing bacteria
 P= 0.88,CI=95%
Fig.S1. Real-time analysis of population of (A) Methanobrevibacter in Healthy vs E. histolytica positive samples.(B) Sulphur reducing bacteria in Healthy vs E. histolytica positive samples. P value =.05 or below was considered significant. CI stands for confidence interval.
